# Spatial variations and socioeconomic determinants of modern contraceptive use in Ghana: A Bayesian multilevel analysis

**DOI:** 10.1371/journal.pone.0230139

**Published:** 2020-03-10

**Authors:** Samuel H. Nyarko

**Affiliations:** 1 Department of Population and Behavioral Sciences, University of Health and Allied Sciences, Hohoe, Ghana; 2 Department of Demography, The University of Texas at San Antonio, San Antonio, TX, United States of America; Anglia Ruskin University, UNITED KINGDOM

## Abstract

The literature on spatial patterns and contextual factors affecting modern contraceptive use is not well-documented in Ghana. This study describes the spatial variations and estimates the socioeconomic determinants of modern contraception among women in reproductive age in Ghana. Using data from the 2014 Ghana Demographic and Health Survey, both global and local Moran’s I test were performed to show spatial autocorrelation and Bayesian multilevel models estimated to determine socioeconomic factors affecting modern contraceptive use. The results show evidence of low prevalence and spatial clustering of modern contraception use across the country. There were also notable regional disparities in contraceptive use that favour mostly the southern regions. Modern contraceptive use is significantly associated with socioeconomic factors such as educational attainment, work status, and marital status, as well as age, religious affiliation, and parity. Contextual factors such as the convenient location of health facility and family planning messages exposure also have a considerable positive effect on modern contraceptive use. Uneducated, unemployed and never-married women are considerably disadvantaged in the utilisation of modern contraception in Ghana. Socioeconomic and contextual factors play a key role in modern contraceptive use in Ghana.

## Introduction

The Sustainable Development Goal 3 aims at ensuring healthy lives and promoting well-being for everybody at all ages. This includes the commitment to ensure universal access to sexual and reproductive healthcare services, including family planning, information and education, and the integration of reproductive health into national strategies and programs by 2030 [1]. Family planning and contraception have proven to have many clear health benefits including the prevention of unintended pregnancies which, in turn, reduces unsafe abortions, maternal morbidity, and mortality among others [2]. Modern contraceptive methods have been said to account for most of the contraceptive use worldwide [3]. In 2012, for instance, contraceptive use is said to have prevented about 218 million unintended pregnancies in developing countries alone and, consequently, prevented 55 million unplanned births, 138 million abortions (40 million were unsafe), 25 million miscarriages and 118,000 maternal deaths [2]. Meanwhile, as of 2017, an estimated 214 million women in developing countries wanted to prevent pregnancy but were not using modern contraception [4].

In western Africa, progress towards the adoption of contraception has been observed to be extremely slow, and attitudinal resistance and limited access to contraceptives are found as the main barriers [5]. Like many developing countries, modern contraceptive use in Ghana is low. For instance, among married women of reproductive age, only a quarter (25%) have been found to use a modern method of contraception [6]. The 1994 Ghana Population Policy and Action Plan puts emphasis on ensuring the availability and accessibility of family planning services to all women and on a voluntary basis, by seeking to accomplish modern contraception prevalence rate of 28% by 2010 and 50% by 2020 [7]. Besides, as part of the global Family Planning 2020 (FP2020) agenda, the Government of Ghana has made major commitments including increasing the demand for family planning considerably by eliminating user fees for family planning services in all public health facilities [8]. Thus, in 2015, the Ghana Family Planning Costed Implementation Plan 2016–2020 was created to accelerate the implementation of the FP2020 commitments as well as align them with important family planning strategies [8]. Despite this, it appears highly unlikely that the 50% by 2020 target will be met owing to several challenges including skilled human resource shortage and financial constraints among others [9].

Quite admittedly, well-documented knowledge about the spatial distribution of modern contraceptive use in Ghana is limited. This paper maps the spatial clustering and disparities in modern contraceptive use among women of reproductive age in Ghana and estimates the individual and aggregate-level factors affecting modern contraceptive use. The paper answers three main research questions: Are there considerable spatial clustering in modern contraceptive use in Ghana? Are there considerable regional variations in modern contraceptive use in Ghana? If yes, do individual-level socioeconomic and contextual factors play a role in the patterns of modern contraceptive use in the country? The answers to these questions have considerable implications for the provision of universal access to sexual and reproductive healthcare services in Ghana. This paper adds spatial and contextual dimensions to the literature on modern contraceptive use in Ghana. Thus, it provides a clear understanding of the spatial dynamics of modern contraceptive use in the country, which is crucial for designing and implementing targeted family planning programs [10].

## Materials and methods

### Data source

This research is based on data from the 2014 Ghana Demographic and Health Survey (GDHS). The 2014 GDHS is the sixth wave in a series of population and health surveys conducted in Ghana as part of the global Demographic and Health Surveys (DHS) Program. The sampling procedure of the survey was based on a multi-stage (two stages) sample design that initially involved selecting clusters that consist of enumeration areas used by the 2010 Population and Housing Census and then the systematic sampling of households [11]. Based on this procedure, a nationally representative sample of 9,396 women aged 15–49, and 4,388 men aged 15–59 were selected from 11,835 households. The data collection instrument comprises three questionnaires–the household questionnaire, the women’s questionnaire, and the men’s questionnaire. The household questionnaire provides a list of all household members while the women’s and men’s questionnaires are used to collect data from women and men, respectively [11]. The unit of analysis comprises 9,396 individual women of reproductive age (15–49 years).

### Study variables and measures

The outcome variable for this study is modern contraceptive use measured at the individual level. This was generated from the variable “contraceptive use and intention” which has four levels–using modern method, using a traditional method, non-user—intends to use later, and does not intend to use. A binary outcome variable was then generated for modern contraceptive use by re-coding “using modern method” as 1 and all other categories as 0.

The main predictor variables used in this study consist of socio-economic factors such as household wealth status (poor, middle, rich), educational attainment (no education, primary, secondary/higher), work status (Working, not working), and marital status (Never married, married/cohabiting, widowed/separated/divorced) measured at the individual level. Also, demographic characteristics such as the age of the woman (<20, 20–29, 30–39, 40–49), parity (0, 1–2, 3–4, 5+), religion (Christianity, Islam, Traditional, Others) and place of residence (urban, rural) were included as control variables. At the aggregate level, a few contextual factors that have the potential to affect modern contraceptive use were also included in the analysis. They include the proportion of health insurance coverage, the proportion of convenient health facility location, health care decision making autonomy and exposure to family planning messages measured as continuous variables at the regional level. These explanatory variables were selected based on the extant empirical literature on the subject.

### Analytic procedure

The data analysis was performed with the R statistical package (version 3.5.2) [12]. To generate descriptive results, a univariate exploratory spatial data analysis (ESDA) was initially performed to show any spatial autocorrelation in modern contraceptive use in the country, using the global Moran’s I statistic. Also, a 4-nearest neighbour weight was set to perform a local Moran’s I test and the fitted values were mapped at the cluster level. Further, a generalised linear model was fitted by region to generate regional modern contraceptive use prevalence and the fitted values were broken using quantile breaks and then merged with a shapefile to produce a map. For the multivariate analysis, Bayesian generalised linear mixed models (Multilevel/Hierarchical) were estimated using the Integrated Nested Laplace Approximations (INLA) approach [13], to examine the effect of socioeconomic characteristics on modern contraceptive use. The Bayesian approach was deployed in this study because it makes more intuitive and meaningful inferences, answers complex questions better and is better suited for decision making compared to the frequentist approach [14]. In Bayesian terms, the effect is modeled as: P(θ| D) ∝ P(D|θ) P(θ); where the posterior distribution is proportional to the likelihood (data) times the prior. Here, uniform (flat) priors were assigned to all the population and regional level parameters. Since the outcome is binary, the multilevel logistic regression is modeled as:

$${\mathrm{logit}(\mathrm{contraceptive}}_{ij})=\beta_{0j}+\sum \beta x_{i}+\gamma z_{j}$$

$$\beta_{0j}=\beta_{0}+u_{j},\;with\;u_{j}∼Normal\;(0,\;\sigma_{u})$$

Where *i* refers to the individual woman, and *j* refers to her region. *β*_*0j*_ is the regional random intercept term while the *βx*_*i*_ term indicates all the individual-level predictors, and the *γz*_*j*_ term is the regression effects for the region-level covariates, while the *uj* is the error term.

Three nested multilevel logistic regression models with the region of residence as the random effect were estimated. While Model 1 was used to estimate the effect of socioeconomic factors, Model 2 was used to control for the effect of demographic factors. Model 3, which is the full model, was used to further control for regional aggregate factors that can potentially influence individual-level contraceptive use. Odds ratios and confidence intervals were then calculated from the posterior means of the model parameters and 95% Bayesian credible intervals. The descriptive analysis was weighted using a complex survey procedure while the multivariate analysis was weighted using individual sample weights. For the complex sampling procedure, the individual sample weights of the respondents are nested in their sample units (clusters) and sample strata (place of residence) to cater for the multi-stage nature of the survey.

## Results

### Descriptive results

Table 1 presents a summary of the results on the background characteristics of the study sample. More than one-third (34.2%) of the study sample were within 20–29 years while one-fifth were within 40–49 years old. The majority (63.1%) of the respondents in the study sample had secondary school education or higher. About 46 percent and 36 percent of them were from rich and poor households, respectively. Over half (56.6%) of the respondents were married or cohabiting and about a third (33.0%) never married. The study sample was also made up of predominantly Christians (80.1%). The majority (73.5%) were employed while the largest proportion (31.3%) of the study sample had 0 parity or had no child at the time of the survey and more than half (53.8%) of them were urban dwellers.

**Table 1:** Background characteristics of respondents.

Variable	Frequency*	Percent
Age of woman		
15–19	1756	17.3
20–29	3135	34.2
30–39	2603	28.4
40–49	1902	20.1
Level of education		
No education	2281	19.1
Primary	1747	17.8
Secondary/higher	5368	63.1
Wealth status		
Poor	4094	35.5
Middle	1902	20.6
Rich	3400	45.9
Marital status		
Never married	3041	33.0
Married/cohabiting	5456	56.6
Separated/divorced/widow	899	10.4
Religious affiliation		
Christianity	7169	80.1
Islam	1726	15.2
Traditional/Spiritualist	226	2.0
Others	274	2.7
Work status		
Working	6761	73.5
Not working	2626	26.5
Parity		
0	2885	31.3
1–2	2557	28.1
3–4	1978	21.6
5+	1976	19.0
Type of residence		
Urban	4602	53.8
Rural	4794	46.2
Modern contraceptive use		
Yes	1735	18.2
No	7661	81.8

*NB: The counts may not match the percentages due to weighting of the percentages

Source: Computed from GDHS 2014 data set

### Spatial variations in modern contraceptive use

**Fig 1:**
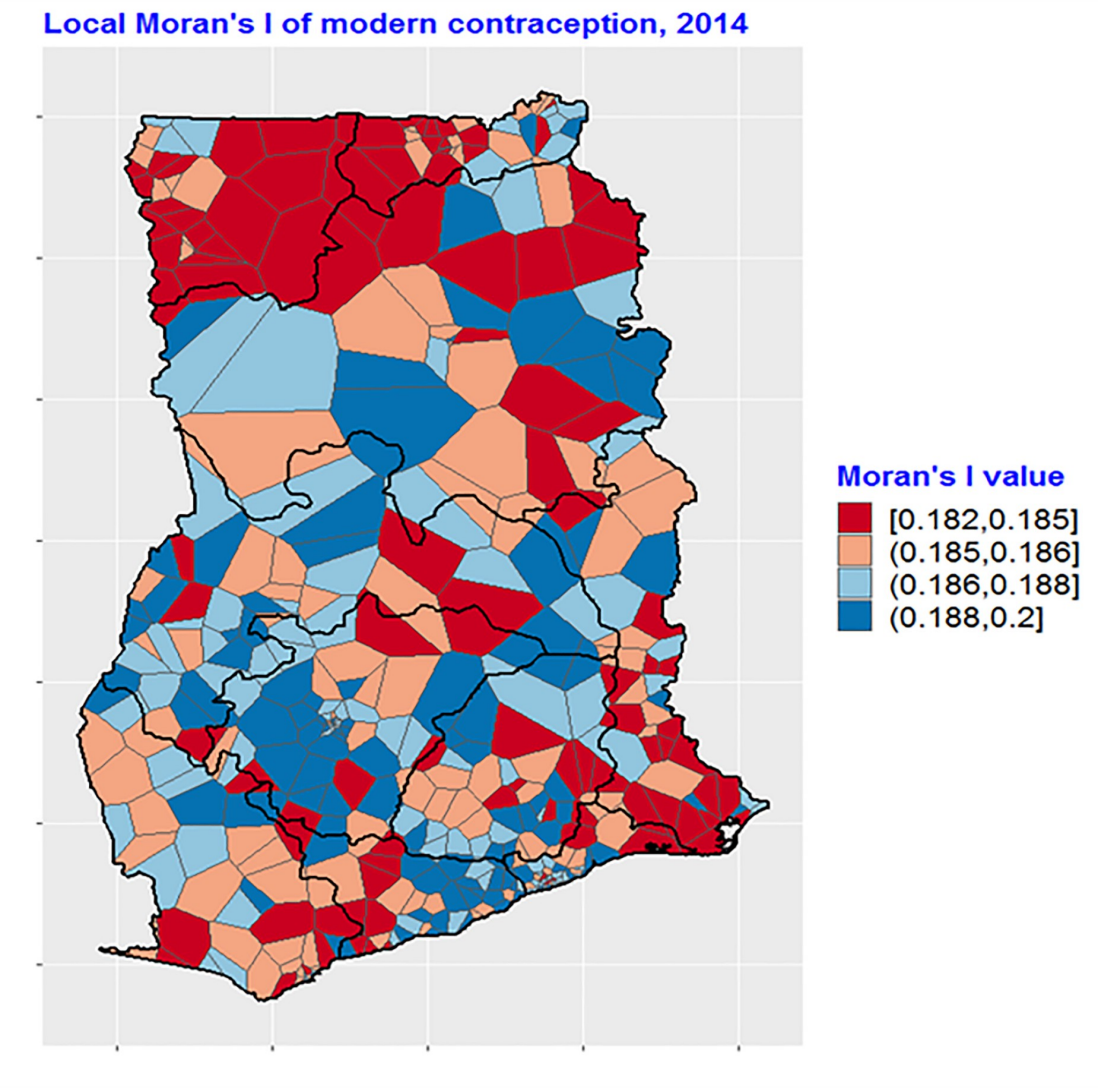
Spatial autocorrelation of modern contraceptive use in Ghana, 2014. Source: Created by the author based on the 2014 GDHS.

The total prevalence of modern contraceptive use was found to be 18.2 percent among the study sample (Table 1). Further, the result of the global univariate Moran’s I test for autocorrelation in contraception showed a significant value of 0.33 (Not shown). Regarding the local Moran’s I test, the results show that there are numerous clusterings in modern contraceptive use across the country (Fig 1). The highest level of clustering ranged between 0.188 and 0.20 showing the blue colouring. Many of these clusterings can be found in the Ashanti region, Brong Ahafo region and around the central portion of the coast as well as some parcels of the Northern region. In contrast, large portions of the Upper East and Upper West regions as well as the southern part of the Volta region show the least clustering in modern contraceptive use (Red colouring).

**Fig 2:**
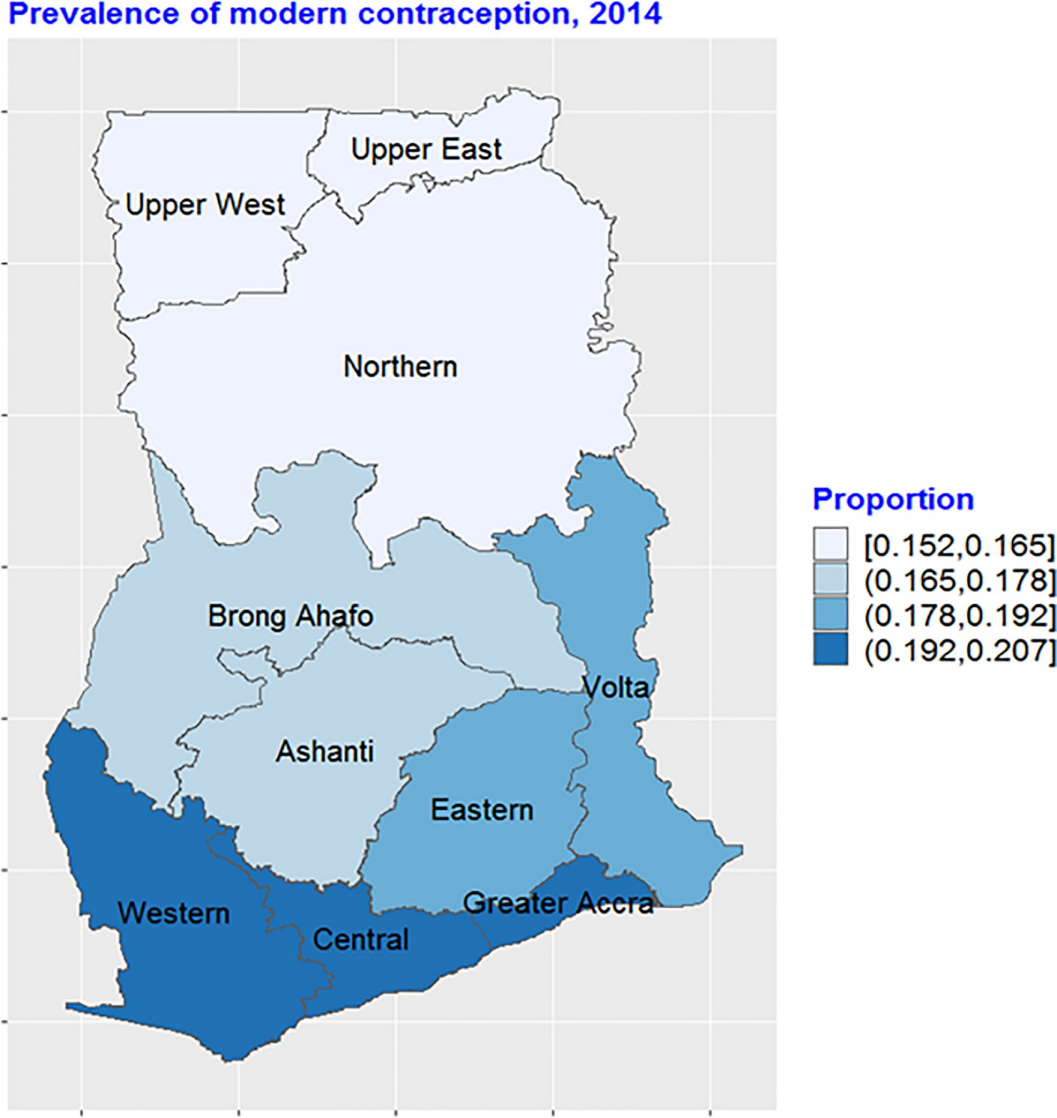
Regional variations in modern contraceptive use in Ghana, 2014. Source: Created by the author based on the 2014 GDHS.

For the regional prevalence, the results show notable inequalities in modern contraception in the country (Fig 2). The highest prevalence of contraception ranged between 19% and 21% showed by the blue colouring whereas the least ranged between 15% and 16.5% represented by the red colouring. It was notable that modern contraceptive prevalence is highest in the southern or coastal regions (Western, Central and the Greater Accra regions) followed by the middle sector regions of the country (Brong Ahafo and Ashanti Regions). The three Northern regions, however, had the lowest prevalence of modern contraception in the country.

### Multivariate analysis results

A summary of the results from the multilevel logistic regression analysis is resented in Table 2. All the models show a similar regional variance of modern contraceptive use of about 0.05. Even though this may seem small, it, however, shows a statistically significant variance in modern contraceptive use at the regional level. In model 1, the socioeconomic factors such as educational attainment, wealth, work and marital status all had an association with modern contraceptive use.

**Table 2:** Bayesian multilevel logistic regression analysis of modern contraceptive use in Ghana, 2014.

Variables	Model 1	Model 2	Model 3
***Socioeconomic factors***	**OR [95% CI]**	**OR [95% CI]**	**OR [95% CI]**
Education (Ref: No education)			
Primary	**1.34[1.13, 1.59]**	**1.37[1.15, 1.64]**	**1.37[1.15, 1.64]**
Secondary/higher	**1.52[1.30,1.78]**	**1.60 [1.35, 1.88]**	**1.60[1.36, 1.89]**
Wealth (Ref: Poor)			
Middle	0.97[0.84, 1.13]	1.02[0.87, 1.20]	1.03[0.87, 1.20]
Rich	**0.80[0.68, 0.92]**	0.98[0.81, 1.18]	0.97[0.80, 1.18]
Work status (Ref: Working)			
Not working	**0.66[0.57, 0.76]**	**0.74[0.64, 0.86]**	**0.74[0.64, 0.86]**
Marital status (Ref: Not in union)			
Married/Cohabiting	**2.31[2.00, 2.67]**	0.97[0.79, 1.19]	0.97[0.79, 1.19]
Separated/divorced/widow	**1.67[1.35, 2.07]**	0.83[0.64, 1.08]	0.83[0.64, 1.08]
***Demographic factors***			
Age (Ref: 15–19)			
20–29		**2.39[1.88, 3.06]**	**2.39[1.88, 3.06]**
30–39		**1.32[1.00, 1.75]**	**1.31[1.00, 1.75]**
40–49		0.87[0.63,1.19]	0.86[0.63,1.18]
Religion (Ref: Christianity)			
Islam		**0.83[0.70, 0.97]**	**0.83[0.70, 0.97]**
Traditional/Spiritualist		**0.43[0.26, 0.67]**	**0.43[0.26, 0.67]**
Others		0.85[0.61, 1.16]	0.85[0.61, 1.17]
Parity (Ref: 0)			
1–2		**2.32[1.86, 2.89]**	**2.33[1.87, 2.91]**
3–4		**4.02[3.11, 5.21]**	**4.06[3.14, 5.25]**
5+		**5.33[4.00, 7.12]**	**5.39[4.04, 7.21]**
Residential type (Ref: Urban)			
Rural		1.11[0.95, 1.28]	1.11[0.96, 1.28]
***Regional contextual factors***			
Percent Health insurance coverage			1.13[0.89, 1.41]
Convenient health facility location			**1.26[1.06, 1.51]**
Family planning message exposure			**1.23[1.00, 1.57]**
Healthcare decision autonomy			0.92[0.77, 1.09]
Region: Variance	**0.06[0.03, 0.20]**	**0.06[0.03, 0.20]**	**0.05[0.02, 0.16]**
WAIC	8656.81	8375.57	8377.21
Log Likelihood	-4379.35	-4285.88	-4304.26s

OR = Odds Ratios, CI = Confidence Intervals; Ref = Reference category; Significant variables are in bold (< 0.05).

However, the effect of wealth status and marital status attenuated considerably after controlling for the demographic and aggregate level factors in models 2 and 3, respectively. On the other hand, educational attainment and work status have maintained their association with modern contraceptive use after taking into consideration the demographic and contextual factors, although the effects slightly waned. In effect, the odds of modern contraceptive use were 37 percent greater for women who had primary school education and 60 percent greater for women with secondary school or higher education compared to women without formal education. Unemployed women had 26 percent lower odds of using modern contraception compared to employed women. Married and cohabiting women had 2.31 odds while separated, divorced and widowed women had 67 percent higher odds of modern contraceptive use compared to never-married women when demographic characteristics were not considered. Unexpectedly, women from rich households had 20 percent lower odds of modern contraceptive use compared to women from poor households when demographic factors are not controlled.

Aside from the socioeconomic factors, the demographic factors such as the age of woman, religious affiliation, and parity had a significant association with modern contraceptive use, but the type of residence had no significant effect. Also, regional level factors such as the convenient location of health facility and family planning message exposure were significantly associated with modern contraceptive use. However, the aggregate effect of health insurance coverage and healthcare decision autonomy of women on modern contraceptive use was found to be weak. The odds of contraception were considerably higher for women aged 20–29 (2.39 times), and 31 percent higher for women aged 30–39 compared to women aged 15–19. The association was, however, weak for women aged 40–49, even though they had lower odds of contraception. Contraception odds were 17 percent lower for Islamic women and 57 percent lower for traditional or spiritual women compared to Christian women. Further, the odds of contraception were substantially higher for women with parity 1–2 (2.33 times), 3-4(4.06 times), and 5+(5.39 times) compared to women with 0 parity or without any child. At the regional aggregate level, a higher proportion of closeness to a health facility led to about 26 percent higher odds of modern contraception. Analogously, a higher proportion of family planning message exposure led to a 23% higher odds of contraception at the individual level. A higher proportion of health insurance coverage increased contraception odds by at least 13 percent at the individual level, even though the effect was weak. Similarly, a higher proportion of women with healthcare autonomy reduced contraception odds by 8 percent at the individual level albeit the effect was not significant.

## Discussion

This study provides empirical information on the spatial variations and patterns of modern contraceptive use in Ghana and how these have been affected by individual-level and contextual factors. The study shows a considerably low modern contraception prevalence among the study sample. In effect, unmet need for contraception is found to play a significant role in unintended pregnancies in Ghana [15]. Furthermore, there is evidence of spatial dependencies or clusterings of modern contraceptive use across the country. The use of modern contraception is highly clustered within some specific parts of the Ashanti and Brong Ahafo Region as well as some parts of the central region. In terms of regional patterns, the findings show evidence of regional inequalities in modern contraceptive use in Ghana. This reflects in both the maps and the multilevel analysis. Some previous studies in Ghana have also shown similar notable inequalities in contraceptive use [16, 17]. In this study, the prevalence of modern contraception is highest in the coastal or southern regions and attenuates as you move northward in the country. Consequently, the three northern regions appear to be the most disadvantaged regions in terms of modern contraceptive use in the country. In essence, this is possibly down to the considerably poorer socio-economic profiles of these regions compared to the southern regions. These regions have the lowest female education and the highest female poverty rate in the country which may underpin their low prevalence of modern contraceptives [18]. A similar situation has been observed in Nigeria where there is a considerable disparity between the north and south in relation to contraceptive use and was found to be explained mainly by socio-economic inequalities as well as ideational factors [19]. There is also notable evidence of spatial disparities in modern contraceptive use in other parts of Africa such as Ethiopia [20], Democratic Republic of Congo [21], and Kenya [22].

Also, in this study, some socioeconomic factors appear to be significantly associated with modern contraception among women of reproductive age in Ghana. For instance, educational attainment has a strong positive association with modern contraception use. Consequently, higher educational attainment becomes a reinforcing factor for modern contraceptive use in Ghana. The positive effect of educational attainment on modern contraceptive use is well-documented in the literature [10, 20, 23]. Higher educational attainment may provide contraception advantage in two major ways. First, educational attainment may provide women with accurate knowledge about contraception, contraceptive options and understand the benefits that can be derived from it. Second, the long duration required by higher educational attainment may in itself encourage sexually active young women to use modern contraception as they seek to postpone pregnancy until after school or perhaps employment.

Furthermore, the findings show that women from rich households are surprisingly associated with reduced odds of modern contraceptive used, but only when demographic characteristics are not considered. This is in sharp contrast with the outcome of previous research in Nigeria [10], as well as Ghana [17] and the Democratic Republic of Congo [21] among others, which show higher modern contraceptive use among the rich households. This may reflect the evidence of increased traditional contraceptive use among women from rich households [11]. The implication may be that some women from rich households may be gradually and subtly shifting from modern to traditional methods, which may have serious implications for modern contraception prevalence in the country.

The findings further show a significant association between the work status of women and modern contraception. This study shows that unemployed women are less likely to use modern contraception compared to their employed counterparts. This provides notable evidence that women's employment is a boost for modern contraceptive use in Ghana which may possibly be down to purchasing power or affordability. Much extant research also corroborates the positive effect of women's employment on modern contraceptive use [20, 23]. This advantage may probably be due to the effect of the affordability of modern contraception services on the part of working women. The findings also indicate that marital status has a considerable effect on modern contraception, only when demographic characteristics and other factors are not considered. In this context, having ever gotten married provides a considerable advantage for modern contraceptive use among women in the study sample [24]. This may have crucial implications for the sexually active unmarried women who find unintended pregnancies and premarital births undesirable.

Additionally, the findings from this research show that some demographic factors play a significant role in modern contraceptive use among women in Ghana. The age of a woman is found to have a significant curvilinear association with modern contraception. The likelihood of modern contraception increases considerably for women aged 20–29 but begins to wane from age 30 and over. The effect of a woman’s age on modern contraception has been shown in the literature [23, 25], even though some studies do not show the same pattern as observed in this study. As well, the findings show a strong association between parity–the number of children ever born by women–and modern contraceptive use. Here, the utilisation of modern contraception increases substantially with the advancing age of the woman. Similarly, higher parity has been widely observed in the literature to be a positive predictor for modern contraceptive use [24, 26]. Quite expectedly, higher parity women may be mainly associated with modern contraceptive use because they may want to stall childbearing.

There is also an indication that religious affiliation affects modern contraception with Christian women being highly associated with modern contraceptive use compared to their counterparts. Similar findings have been previously documented in Ghana [16] and Nigeria [27]. Thus, being a Muslim woman and other religious affiliate provides a considerable disadvantage in the utilisation of modern contraceptives [27].

Moreover, this study provides evidence of some regional contextual effects on modern contraceptive use among women of reproductive age in Ghana. Closeness to health facilities plays a considerably positive role in modern contraceptive utilisation at the regional level. Women residing in regions with a lower proportion of health facilities located at a convenient distance face a substantial disadvantage in the utilisation of modern contraception in Ghana. The effect of physical access to health facilities has also been evident in Kenya [25] among other developing countries. This disadvantage has crucial implications for contraceptive use rate as well as the level of unintended pregnancies and induced abortions in Ghana. It is also evident that a higher proportion of regional family planning message exposure has a considerable positive effect on modern contraceptive use in the country. Increased exposure to family planning messages at the regional level enhances the utilisation of modern contraception by at least 23 percent. Hence, extensive provision of family planning education will provide a substantial boost in the utilisation of modern contraception in Ghana. This affirms findings of similar studies in Kenya [25] and sub-Saharan Africa [28] at large, which also show a significant positive effect of family planning messages on modern contraceptive use.

## Conclusions

The findings show that modern contraceptive use is low in Ghana. Modern contraceptive use is usually clustered within some particular regions across the country. Regional disparities in modern contraceptive prevalence are notable in the country with the northern regions being at a substantial disadvantage compared to the southern sector regions. Individual-level socioeconomic and demographic characteristics, as well as some regional contextual factors such as the convenience of health facility location and family planning message exposure, play a major role in modern contraceptive prevalence in Ghana. Uneducated women, unemployed women, and never-married women are highly disadvantaged in modern contraceptive use in Ghana. This also applies to younger women, Islamic and traditional or spiritualist women, as well as lower parity women. This research underscores the Sustainable Development Goal 3 and reinforces the necessity to improve modern contraceptive use by providing universal access to family planning services, information and education to women in regions that lack them. Ultimately, substantial improvement in modern contraceptive use should hinge on abridging socioeconomic inequalities among women in Ghana, with the uneducated, unemployed and never-married women being the major targets of family planning programs. The findings further show the need for region-specific family planning programs based mainly on prevailing contextual factors.
